# A meta-analysis of HDL cholesterol efflux capacity and concentration in patients with rheumatoid arthritis

**DOI:** 10.1186/s12944-021-01444-6

**Published:** 2021-02-21

**Authors:** Binbin Xie, Jiang He, Yong Liu, Ting Liu, Chaoqun Liu

**Affiliations:** 1 Department of Nutrition, School of Medicine, Jinan University, Guangzhou, 510632 Guangdong China; 2 Department of Mathematics and Physics, School of Biomedical Engineering, Southern Medical University, Guangzhou, 510515 Guangdong China; 3 Department of Laboratory Medicine, Hospital of Stomatology, Anhui Medical University, Hefei, 230032 Anhui Province China

**Keywords:** Rheumatoid arthritis, Cholesterol efflux capacity, High-density lipoprotein, Meta-analysis

## Abstract

**Background:**

Poor cholesterol efflux capacity (CEC) has been proposed to be an independent risk factor for cardiovascular diseases. However, current evidence is inconsistent, especially in rheumatoid arthritis (RA) patients. This meta-analysis aims to identify whether CEC is impaired or altered by drug therapy in RA.

**Methods:**

The PubMed/MEDLINE, Embase, Cochrane Library and ClinicalTrials.gov databases were browsed to identify studies on CEC in RA patients. The searches mainly focused on studies in human subjects that were published before November 14, 2020, without any language restrictions. The effect size was pooled by the standardized mean differences and mean differences (SMD & MD) as well as the corresponding 95% confidence intervals (CIs) in a random or fixed effect model. Heterogeneity across the studies was tested using Cochran’s Q test and *I*^*2*^ statistic. Newcastle-Ottawa Scale and the Downs and Black scale (D&B) were applied to evaluate the quality of included studies. The GRADE-system with its 4-grade evidence scale was used to assess the quality of evidence.

**Results:**

A total of 11 eligible articles, including 6 observational and 5 interventional studies, were retrieved. The pooled results showed that in patients with RA, CEC was not significantly different than in healthy controls (SMD: -0.34, 95% CI: − 0.83 to 0.14), whereas the plasma HDL-C levels was significantly lower (MD: -3.91, 95% CI: − 7.15 to − 0.68). Furthermore, in the before-after studies, the CEC of RA patients (SMD: 0.20, 95% CI: 0.02 to 0.37) increased, but the plasma HDL-C levels (MD: 3.63, 95% CI: − 0.13 to 7.39) remained at a comparable quantity after anti-rheumatic treatment comparing with the baseline. In addition, the funnel plot of included studies displayed a lightly asymmetry, while Egger’s and Begg’s test did not suggest the existence of publication bias. The quality of evidence was rated according to GRADE as moderate to very low.

**Conclusion:**

The current meta-analysis demonstrated that HDL-mediated CEC can be improved by the early control of inflammation and anti-rheumatic treatment in RA patients, which is independent of the plasma HDL-C levels. However, the results should be interpreted with caution because of low-quality and limited quantity of evidence. Future randomized controlled trials are needed to determine whether therapeutic strategies to enhance CEC in RA patients have beneficial effects for preventing CVD.

**Supplementary Information:**

The online version contains supplementary material available at 10.1186/s12944-021-01444-6.

## Introduction

Rheumatoid arthritis (RA), a chronic polyarthritis autoimmune disease that causes arthrosis impairment and even leads to functional disability [1], affects approximately 0.3–1.0% of people worldwide [2]. RA gives rise to a heavy burden for both patients and society, and those living with RA have a significantly shorter life expectancy. A higher risk of cardiovascular diseases (CVDs) has been found in patients with RA than those in the general population [3]. RA-induced inflammation increases arterial stiffness, changes the lipid profile, and destabilizes plaques. Moreover, 60% of excess mortality among RA patients is attributed to CVDs [4], which is one of the most severe complications of RA and cannot be fully explained by traditional cardiovascular risk factors.

Researches showed that dyslipidemia involving low HDL-C and high low-density lipoprotein cholesterol (LDL-C) is a major risk factor of cardiovascular events [5–7]. Numerous studies have demonstrated that plasma HDL-C levels are also lower in the RA patients than those in the general population [8]. For instance, a study noted that an average 9% decrease in plasma HDL-C levels has been observed before the onset of symptoms among RA patients [9]. In addition, paradoxical associations among low lipid levels (i.e., total cholesterol (TC), LDL-C and HDL-C levels) and the ongoing risk of CVDs have been observed in patients with poorly controlled RA [10], while initial reductions in these parameters have been shown to increase along with anti-inflammatory treatment in patients with RA. Recent clinical trial has revealed that HDL-C-raising therapies do not considerably attenuate the risk of cardiovascular events in individuals at high risk [11]. Therefore, whether the concentrations of circulating HDL-C in patients with RA change even after anti-inflammatory treatment merits further investigation.

HDL possesses several key atheroprotective functions, including reverse cholesterol transport, endothelial function maintenance, anti-inflammatory activity and platelet aggregation inhibition [12]. Among these functions, reverse cholesterol transport (RCT) refers to an overriding process that promotes excess plasma cholesterol from the cell to the liver for catabolism [8]. Macrophage cholesterol efflux is the first critical step of RCT and considered as a key atheroprotective property [13]. The general methods for measuring cholesterol efflux capacity (CEC) include using radioisotope-labeled cholesterol that is labeled with [^3^H]-cholesterol ([^3^H]-C) and fluorescence-labeled cholesterol to measure CEC [14, 15]. Impaired CEC has been proven to increase the risk of CVDs, which is independent of the plasma HDL-C levels [16]. However, the epidemiological data that have been used to explore the association between CEC and RA are inconsistent. Some of the previous studies have shown that the CEC is significantly lower in RA patients than those in healthy controls, while others have failed to reach conclusions [17–26].

Therefore, in order to assess the conflicting results, the epidemiological changes in CEC as well as plasma HDL-C levels among RA patients were systematically reviewed and meta-analyzed.

## Methods

### Literature search and selection criteria

This study has been registered in PROSPERO database and the registration number is CRD42020209010. This meta-analysis was conducted and written according to the PRISMA guidelines. PubMed/MEDLINE, EMBASE, Cochrane Library and ClinicalTrials.gov databases were browsed and searched for studies reporting CEC in patients with RA. Searches focused on human subjects, with no restriction on languages, and published before November 14, 2020. To avoid missing any relevant studies, the references of identified studies and review papers were manually explored. The following medical subject headings terms and keywords were used alone or in combination: (rheumatoid arthritis OR RA OR rheumatoid arthritis [MeSH]) AND (cholesterol efflux OR HDL-mediated cholesterol efflux OR reverse cholesterol transport OR cholesterol efflux capacity) AND (HDL-C OR HDL OR high density lipoprotein OR high density lipoprotein [MeSH]). Details of the searching strategy are available in the online Additional file.

### Study selection

The studies were initially assessed according to the following inclusion criteria: 1) intervention and observational studies; 2) studies including only subjects older than 18 years of age; 3) studies including RA patients who fulfilled the 1987 or 2011 the American College of Rheumatology (ACR) criteria, regardless of disease severity or concomitant diseases; 4) studies including healthy control subjects without inflammatory conditions; and 5) studies comprising CEC and HDL-C levels as the outcomes of interest. The exclusion criteria were as follows: 1) studies that did not report CEC and HDL-C levels; 2) studies with a repeated study population; and 3) animal research, letters, or meeting abstracts.

Two researchers independently removed the duplicate records and screened the titles and abstracts to identify the potentially relevant articles. Another two researchers independently screened the full texts to identify additional eligible studies. If the data were duplicated in more than one study, the study with the largest dataset and population was selected for inclusion.

### Data extraction and outcome measures

Two reviewers independently extracted data on each eligible article by using a standardized data collection form, including the first author’s name, publication year, study design, country, patient characteristics, sample size, sex distribution, CEC assay methods, plasma HDL-C levels, CEC, disease activity score for 28 joints (DAS28), C-reactive protein (CRP), erythrocyte sedimentation rate (ESR), records of all medicines taken, length of the follow-up period and study outcome. Although some participants were followed-up for varying durations, data were extracted for the longest follow-up only. If necessary, the authors of the studies were contacted for additional data. Disagreements between reviewers were resolved by discussion with a third author.

### Quality and risk assessment

Two quality rating scales were used to assess the methodological quality of the studies. The quality of the observational studies was evaluated by the Newcastle-Ottawa Scale (NOS) [27]. According to the guidelines, three aspects were assessed: selection, comparability and exposure. Scores of 1–4 were defined as low quality, while scores of 5–9 were defined as high quality. On the other hand, the Downs and Black (D&B) scale was adopted to analyze the risk of bias in nonrandomized and randomized studies from a list of 27 criteria [28]. The last question about power was replaced by a modified version that was published in the previous systematic reviews [29]. Scores of 1–14 were defined as low quality, and scores of 15–32 were defined as high quality.

### Outcome measures

The primary outcome measures were the levels of CEC and HDL-C. CEC was measured by using a donor cell, which can release a cholesterol tracer and a cholesterol acceptor. HDL-C was measured directly in serum or plasma. The secondary outcome measures were CRP and ESR, LDL-C, TC and triglyceride (TG). ESR as a marker of inflammatory activity was added for the current study, which was different with the original protocol.

### The assessment of cumulative evidence

The grading of Recommendations Assessment, Development and Evaluation (GRADE) methodology was applied to evaluate the quality of accumulative evidence [30]. Per GRADE, randomized trials begin as higher quality evidence and the quality may be downgraded based on limitations, inconsistency, indirectness, imprecision, and publication bias. Evidence from non-randomized studies starts as low-quality evidence and can be upgraded in consideration of three factors: large magnitude of effect, evidence of a dose-response effect, and all plausible confounders. The GRADE approach ranks quality of evidence into four grades: high, moderate, low and very low. The GRADE assessment was performed using GRADEpro software (McMaster University, Hamilton, Canada) [31].

### Statistical analysis

#### Assessment of summary effect and heterogeneity

The data from the observational studies and intervention studies were analyzed. Mean and standard deviations (SDs) were used to evaluate the changes in CEC and plasma HDL-C levels in RA patients. If data were presented as median (interquartile range) or mean ± standard error, mean and standard deviation were calculated from these values by using Hozo’s approach [32]. The measurement of lipoprotein-lipid parameters originally reported as millimoles were converted to milligrams per decilitre by using an online unit conversion tool (www.onlineconversion.com/cholesterol.htm) for calculation. According to the Cochrane handbook, the standardized mean differences and mean differences (SMD & MD) as well as the 95% confidence intervals (CIs) are regularly used for meta-analysis of continuous data [33]. SMD was employed when different instruments were used to measure the same underlying construct. Otherwise, MD was chosen to synthesize data. For the intervention studies, the baseline data was recorded as the control group, and the data from the end of the treatment phase was collected as the experimental group. Heterogeneity among the included studies was observed by using Cochran’s Q test and *I*^*2*^ statistic. When heterogeneity was low (*I*^*2*^ < 50%) and *P* < 0.05, the fixed effect model would be applied to synthesize the data, while *I*^*2*^ > 50% and *P* > 0.05, the random effect model will be used.

### Estimation of prediction intervals

Prediction intervals (PIs) represents the range of true effects in future studies which will be broader than the confidence interval in case of high heterogeneity. To further account for heterogeneity between studies, 95% PIs was estimated for the summary random effect estimates, assuming true effect sizes are normally distributed [34, 35].

### Credibility ceiling test

The credibility ceilings test was performed to account for potential methodological limitations of observational studies that might lead to spurious precision of summary effects due to unmeasured confounding factors and biases. The outcome of each observational study was assumed to have a probability c, called credibility ceiling, that the true effect size is on the opposite direction of the effect suggested by the point estimate. A probability was calculated, and compared with a predefined credibility c%. A variance will be recalculated if the probability is less than credibility c% [36]. In this study, the level was set at 10% to re-estimate the inter-study heterogeneity and the pooled effect size.

### Subgroup analysis and sensitivity analysis

Subgroup analysis was additionally conducted to determine the sources of heterogeneity according to the mean age of participants, DAS28 and study design. However, there are some deviations from the study protocol in subgroup analysis. Analyses stratified by BMI and gender was not performed, because the subtle difference of BMI as well as some mixed gender groups among the included studies failed to reach stratification criterion. If subgroup analysis showed statistically significant *P* values for interaction (*P* < 0.05), suggesting that the overall effect was different across certain subgroups. Sensitivity analysis was subsequently conducted by comparing two different models to enhance the robustness of the results. Besides, robustness of the results was assessed by excluding one study at a time from the pooled analyses. The risk of publication bias was examined through visual inspection of funnel plots and Egger’s and Begg’s test, where *P* < 0.1 indicated potential publication bias [37, 38]. For the missing data, authors were contacted for additional information.

All analyses were implemented in STATA, version 14.0 (StataCorp. 2015. Stata Statistical Software: Release 14. College Station, TX: StataCorp LP.), R version 3.6.3 and Review Manager, version 5.3 (Copenhagen: The Nordic Cochrane Centre, The Cochrane Collaboration, 2014).

## Results

### Literature search results and study characteristics

**Fig. 1:**
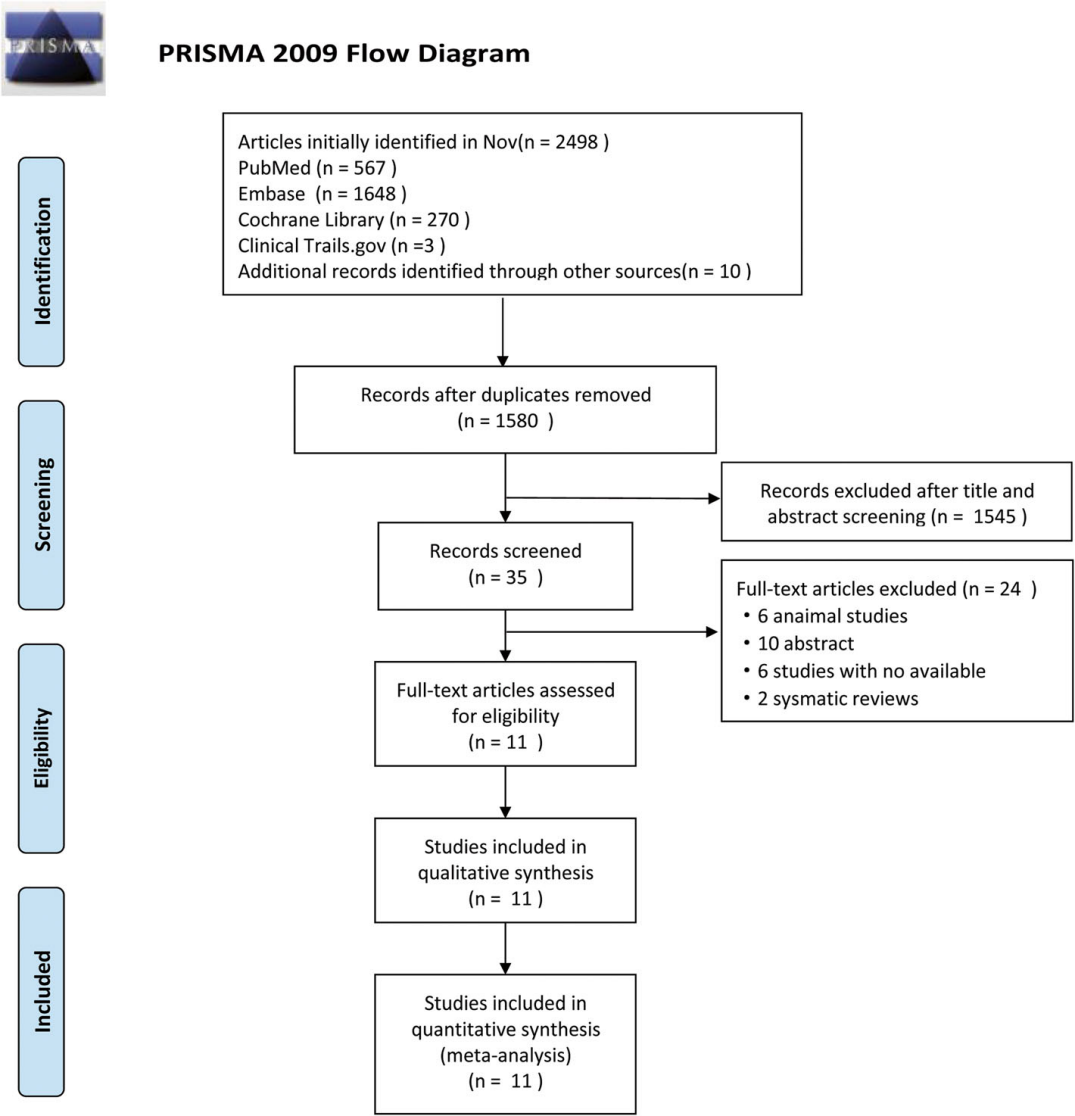
Flow diagram of selection process in the meta-analysis

A total of 2498 articles were initially retrieved from the various databases. After removing duplicated articles and screening the titles and abstracts, 2463 articles were further excluded. The full texts of the remaining 35 articles were reviewed according to the inclusion criteria. Eventually, only 11 articles were included. 26 articles were excluded for the following reasons: 6 studies did not assess the outcome of CEC; 3 were reviews; 10 were abstracts of articles for which the full texts were not available; and 6 were animal studies. For the rest of the studies listed, there were a total of 6 observational studies and 5 intervention studies. The whole screening process is shown in the flow chart in Fig. 1.

Table 1 and Table 2 show the main characteristics of the included studies. These 11 articles were published from 2012 to 2019. Of these studies, 7 were conducted in the USA, 3 were conducted in the UK, and 1 was conducted in Spain. The sample size of these studies ranged from 36 to 401; several of them had a sample size ranging from 50 to 100, which was considered as a modest size, and 3 other studies had a large sample size (> 100). The average age of the participants ranged from 42 to 65 years. Cases and controls were matched by gender, age and body mass index (BMI) in the case-control studies. According to the NOS and D&B standard criteria, the quality scores of the 6 observational studies ranged from 5 to 8, which indicated moderate to high quality. Moreover, the quality scores of the intervention studies ranged from 11 to 17, and 2 studies were considered as high-quality and 3 were considered as low-quality studies. Most observational studies did not report the nonresponse rate, and the D&B scores indicated that the intervention studies had weak external validity.

**Table 1:** Characteristics of observational studies in this systematic review and meta-analysis

Authors	Country	Age (mean)	Study type	Subjects (female, %)	Duration^a^ (month)	Assay method of CEC	Labeled-cholesterol	Quality score	Outcome summary	BMI
Ronda et al., 2013 [17]	USA	45.0	Case-control	52 (86.7)	NA^c^	J774, Fu5AH, CHO-k1 to ApoB-depleted serum	^3^H-cholesterol	5	CEC^b^ is impaired in RA	NA
Vivekanandan-Giri et al., 2013 [18]	USA	56.6	Case-control	34 (58.6)	4.0	J774 to ApoB-depleted serum	^3^H-cholesterol	5	CEC was diminished in RA patients	Case: 27 ± 5 Control: 27 ± 7
Charles-Schoeman et al., 2015 [19]	USA	54.0	Case-control	66 (82.0)	12.8	RAW264.7 to isolated HDL	^3^H-cholesterol	9	no difference of CEC between RA patients and controls	Case: 28 ± 7 Control: 25 ± 4
O’Neill et al., 2016 [20]	UK	59.1	Case-control	22 (73.0)	5.0	J774 to ApoB-depleted serum	^3^H-cholesterol	7	–	Case: 24 ± 4 Control: 26 ± 2
Ormseth et al.,2016 [21]	USA	54.0	Cross-sectional	144 (68.6)	3.9	THP-1 to ApoB-depleted serum	^3^H-cholesterol	6	CEC is not significantly altered in RA patients	Case: 28 (24, 33) Control: 27 (25, 32)
Tejera-Segura et al., 2017 [22]	Spain	57.2	Cross-sectional	295 (73.6)	7.0	J774 to ApoB-depleted serum	BODIPY-cholesterol	7	CEC is not significantly altered in RA patients	Case: 28 ± 5 Control: 28 ± 5

Data are presented as median (interquartile range) or mean ± SD unless otherwise indicated; ^a^ duration of RA; ^b^ cholesterol efflux capacity; ^c^ not available

**Table 2:** Characteristics of interventional studies in this systematic review and meta-analysis

Authors	Country	Treatment	Dose of drugs	Study design	N/female (%)	Mean (age)	Duration^a^ (year)	Length of follow-up (m)	Quality score	Outcome summary	BMI
Ronda et al., 2015 [23]	USA	MTX^c^, ADA^d^, MTX	MTX: 3–5 mg ADA: 40 mg	One-arm study	56 (NA)	57.0	2.0	6	11	CEC^b^ was not significantly altered after RA therapy	NA
P.Liao et al., 2015 [24]	USA	MTX, INF^e^, MTX + INF	NA^i^	One-arm study	80 (88.9)	57.0	16.5	12	13	CEC was improved in CEC after therapy	Baseline: 27 ± 5
O’Neill et al., 2016 [20]	UK	MTX + INF, MTX + PLA^f^	M + I: 5 mg/kg M + p: 2.5–25 mg/kg	Two-arm study	18 (66.7)	58.6	5.0	12	16	CEC was not significantly altered after RA treatment	Treatment: 25 ± 5 placebo: 25 ± 1
Ormseth et al., 2016 [25]	USA	MTX, TCZ^g^, ADA^h^	NA	Three-arm study	59 (84.0)	53.0	9.0	6	17	CEC was not significantly altered after RA treatment	NA
Ferraz-Amaro et al., 2019 [26]	UK	TCZ	8 mg/kg	One-arm study	24 (88)	52.0	8.0	12	12	CEC was significantly increased after RA treatment	Treatment: 29 ± 6 Baseline: 28 ± 5

Data are presented as median (interquartile range) or mean ± SD unless otherwise indicated; ^a^ duration of RA; ^b^ cholesterol efflux capacity; ^c^ methotrexate; ^d^ adalimumab; ^e^ infliximab; ^f^ placebo; ^g^ tocilizumab; ^h^ adalimumab; ^i^ not available

### The main outcome: changes in the CEC and HDL-C levels among RA patients

**Fig. 2:**
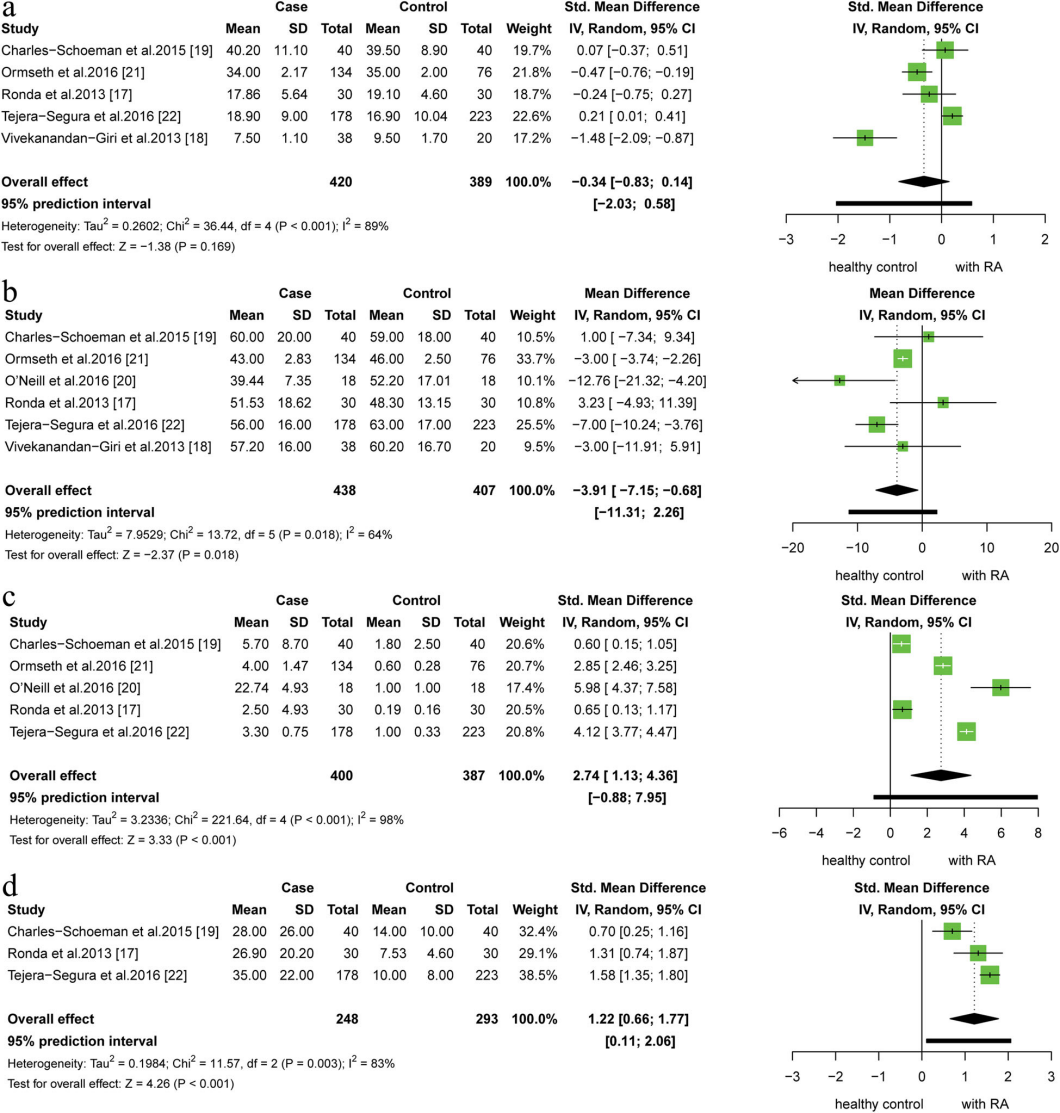
Forest plot of the plasma levels of CEC (a), high-density lipoprotein (b), C-reaction protein (c) and erythrocyte sedimentation rate (d) for patients with RA versus control group in observational study

A total of 5 observational studies reported the CEC levels in both case and control groups, with a total of 809 participants. The pooled results showed that the level of CEC of RA patients was not significantly lower than that of healthy controls (SMD: -0.34, 95% CI: − 0.83 to 0.14; 95% PI: − 2.03 to 0.58; *I*^*2*^ = 89%, *P* for heterogeneity < 0.001). In addition, 6 studies including 845 subjects measured the plasma HDL-C levels showed that patients with RA had lower HDL-C levels (MD: -3.91, 95% CI: − 7.15 to − 0.68, 95% PI: − 11.31 to 2.26; *I*^*2*^ = 64%, *P* for heterogeneity = 0.018) (Fig. 2). The plasma TC and LDL-C levels were significantly lower between the two groups, but not for TG levels. (see Additional file 1).

**Fig. 3:**
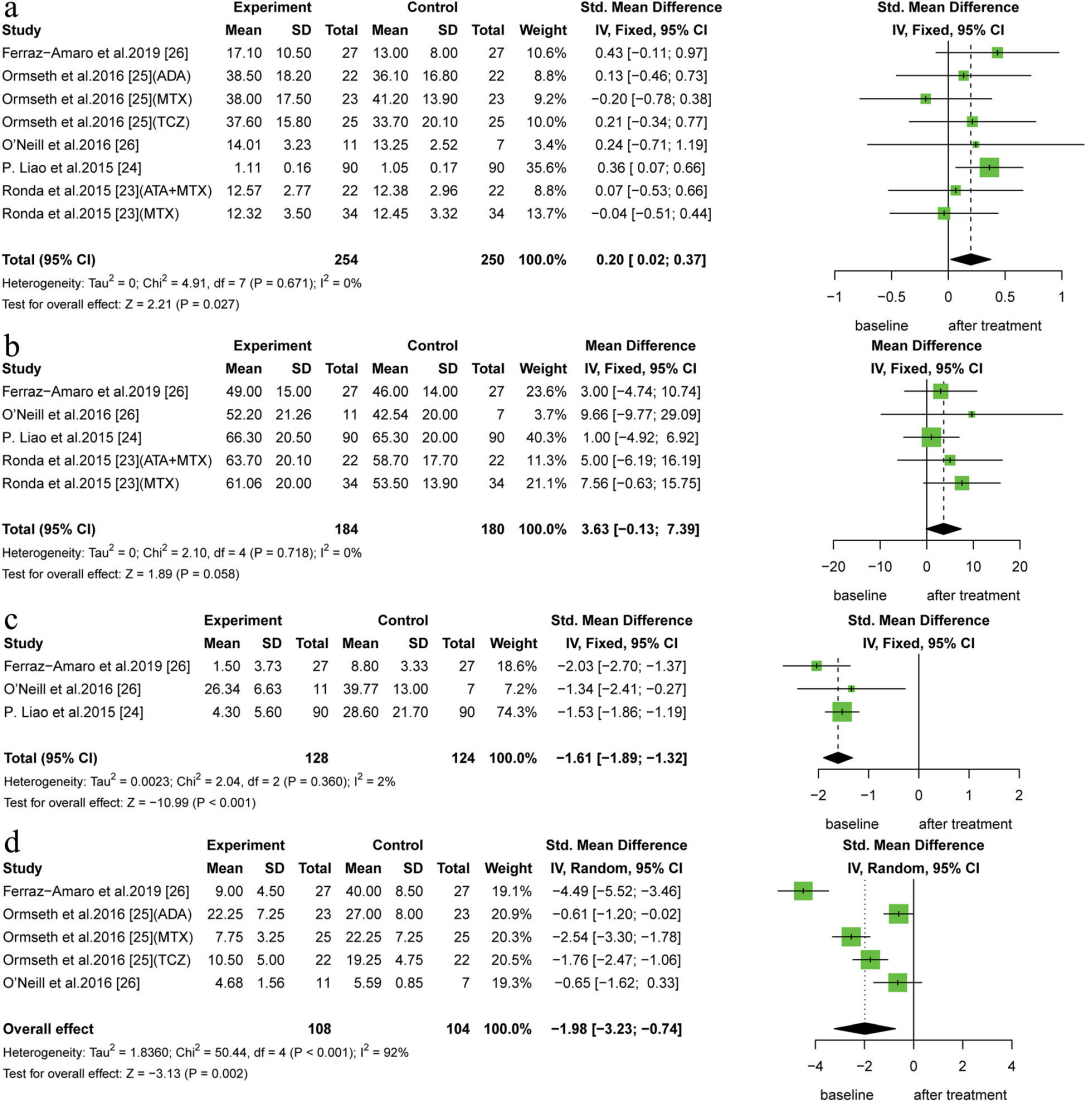
Forest plot of the plasma levels of CEC (a), high-density lipoprotein (b), C-reaction protein (c) and erythrocyte sedimentation rate (d) for patients with RA and control group in before-after studies

A total of 5 before-after studies including 8 trials revealed a statistically significant elevation of CEC in RA patients who had taken anti-rheumatic medications, comparing to the baseline (SMD: 0.20, 95% CI: 0.02 to 0.37; *I*^*2*^ = 0%, *P* for heterogeneity = 0.680). In addition, only 4 intervention studies were well qualified for inclusion in the analysis of the change in HDL-C levels. The plasma HDL-C levels was increased in the RA patients after anti-rheumatic drug treatment, comparing to the baseline (MD: 3.63, 95% CI: − 0.13 to 7.39). The heterogeneity was quite high (*I*^*2*^ = 0%, *P* for heterogeneity = 0.640) (Fig. 3). Other lipid parameters, such as LDL-C, TC, and TG levels, were not significantly different between the baseline and follow-up (see Additional file 2).

### The secondary outcome: the CRP and ESR measurements in RA

In the observational studies, 5 studies reported the CRP level as a continuous variable. A pooled analysis of data showed that the CRP level was higher in the RA patients than that in the healthy controls (SMD: 2.74, 95% CI: 1.13 to 4.36; 95% PI:-0.88 to 7.95; *I*^*2*^ = 98%, *P* for heterogeneity < 0.001). (Fig. 2). However, the CRP level significantly decreased after anti-rheumatic drug therapy in patients with RA in the interventional studies (SMD: -1.61, 95% CI: − 1.89 to − 1.32; *I*^*2*^ = 2%, *P* for heterogeneity = 0.360). High level of ESR has been found in patients with RA (SMD: 1.22, 95% CI: 0.66 to 1.78; 95% PI: 0.11 to 2.06; *I*^*2*^ = 83%, *P* for heterogeneity = 0.003), while the pooled SMD from 5 trials revealed ESR levels was decrease in RA patient who received anti-inflammation therapy (SMD: -1.98, 95% CI: − 3.23 to −.0.74; *I*^*2*^ = 92%, *P* for heterogeneity < 0.001) (Fig. 3).

### Subgroups and sensitivity analysis

Substantial heterogeneity was observed in the meta-analysis. Analyses stratified by age (< 55 years / ≥ 55 years) and study design were inconsistent in the overall effect size of HDL (age: *P* < 0.001 vs. *P* = 0.122; study design: *P* = 0.430 vs. *P* = 0.018). However, no significant differences were found after stratification by DAS28. Part of the heterogeneity might be explained by various age groups and the type of study design (see Additional file 3-5). Sensitivity analysis showed that the overall effect sizes of CEC, CRP and ESR obtained using the fixed-effects and random-effects models were identical, and no single study brought significant effect on the pooled results. When two studies were excluded from the meta-analysis [21, 22], however, the result of HDL-C was different from the previous conclusion. (see Additional files 6 and 7). Two studies had a substantial impact on the robustness of the overall estimate by excluding one study at a time. The credibility ceiling test was further used to conduct sensitivity analysis for four outcomes. In 10% credibility ceiling, the results of ESR and CRP retained statistical significance (ESR, SMD: 0.93, 95% CI: 0.05 to 1.81, *I*^*2*^ = 0; CRP, SMD: 0.74, 95% CI 0.08 to 1.40, *I*^*2*^ = 0), while HDL-C lost statistical significance in this analysis (MD: -2.90, 95% CI:-5.24, 1.05, *I*^*2*^ = 0). The pooled estimate of CEC was − 0.04 (95% CI: − 0.33 to 0.25) with estimate *I*^*2*^ = 31.8%. (see Additional file 8).

### Publication bias

**Fig. 4:**
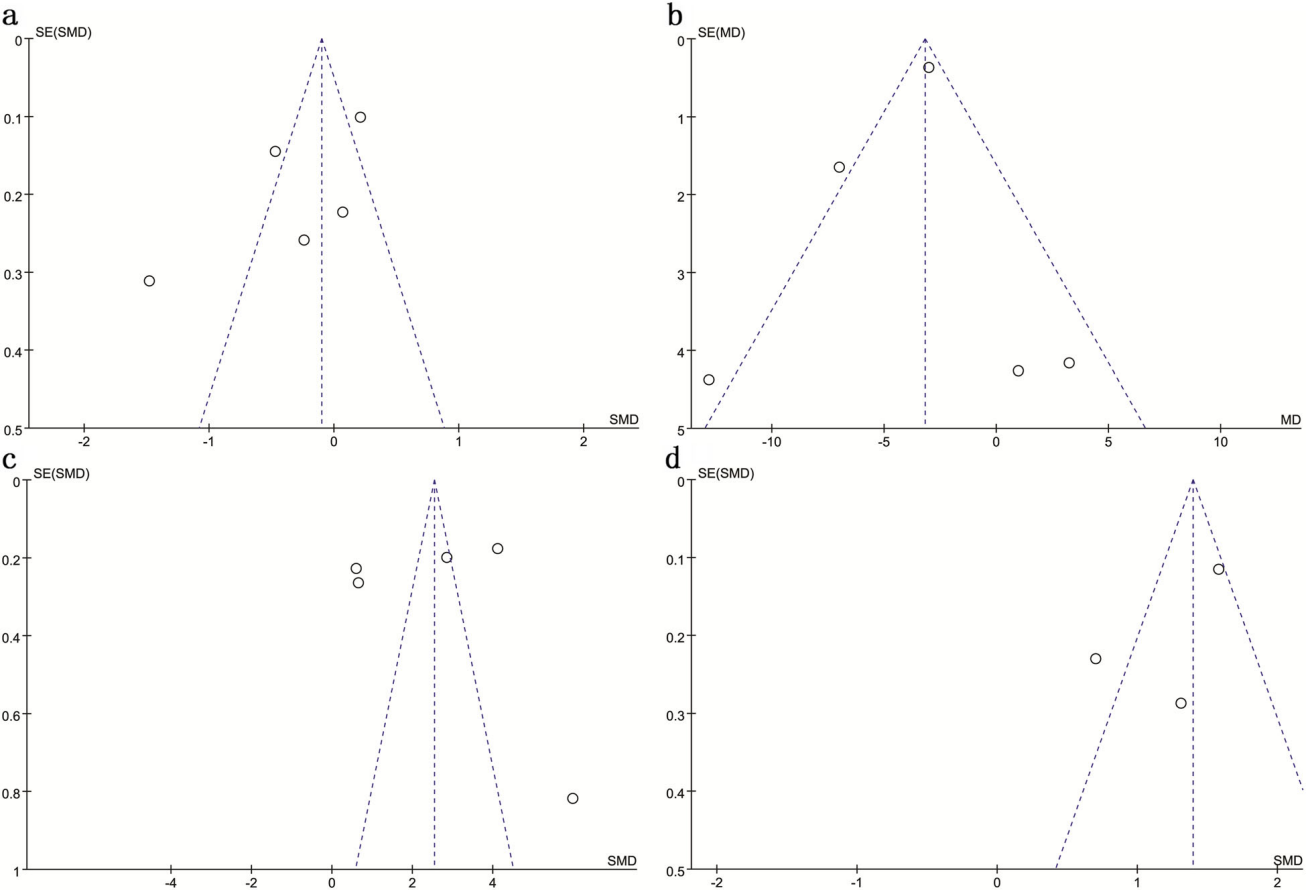
Funnel plots evaluating the pooled estimates for the plasma levels of CEC (a), high-density lipoprotein (b), C-reaction protein (c) and erythrocyte sedimentation rate (d) among patients with RA.

The funnel plots for CEC, HDL, CRP and ESR were created to assess the publication bias (Fig. 4). The Begg’s and Egger’s tests were also used to evaluate the publication bias. Currently, the results of Egger’s test (*P*_*CEC*_ = 0.201; *P*_*HDL-C*_ = 0.699; *P*_*CRP*_ = 0.932; *P*_*ESR*_ = 0.475) and Begg’s test (*P*_*CEC*_ = 0.221; *P*_*HDL-C*_ = 0.452; *P*_*CRP*_ = 0.806; *P*_*ESR*_ = 1.000) revealed no significant publication bias. However, the statistical power for detecting publication bias was low due to the small number of studies.

### The assessment of cumulative evidence

The GRADE rating for the quality of evidence on each outcome parameter was shown in Additional files 9 and 10. In observational studies, the outcomes of CEC, CRP and ESR were judged to very low evidence, downgrading for inconsistency of results. HDL-C was judged to low. In interventional studies, evidence quality ranged from moderate to very low, which may allow direct comparisons of treatments. For CRP, the quality of the evidence was assessed as moderate due to limitation of study, while CEC and HDL-C were deemed as low because of limitation of study and imprecision, and ESR was deemed as very low due to inconsistency and imprecision.

## Discussion

To the best of the author’s knowledge, this is the first systematic review and meta-analysis to simultaneously evaluate whether the CEC and plasma HDL-C levels are decreased or altered by drug therapy in individuals with RA. In summary, the evidence revealed that no significant difference was found with respect to serum HDL-meditated CEC levels, while the plasma HDL-C level was significantly lower in the patients with RA than those in the healthy subjects. A decrease of CRP and ESR were found in RA patients compared with healthy control. In addition, stratified analysis by age and study design could partially explain the moderate heterogeneity of HDL-C. However, it is interesting that in the interventional studies, CEC was significantly elevated in RA patients who were receiving medications, as well as a reduction in the CRP levels as compared to the control baseline. These findings suggested that the inhibition of inflammation might as well improve HDL-mediated CEC. Although the plasma HDL-C levels increased slightly after RA treatment, the difference was not statistically significant, which indirectly indicated that CEC was more sensitive than HDL-C levels. However, the GRADE assessment revealed that evidence quality ranged from moderate to very low. Thus, interpretation of the results must be conducted with caution and further randomized controlled studies are warranted to confirm the role of HDL-C function and concentration in RA.

In 2012, Charles-Schoeman et al. was the first to report that there was no significant difference in CEC between RA patients and control subjects, despite CEC was shown to be inversely associated with high disease activity in RA patients [19]. Subsequently, another case-control study published in 2013 that analyzed different CEC efflux pathways demonstrated that ABCG1-mediated CEC was remarkedly impaired in individuals with RA [17]. In 2015, another study conducted by Ronda et al. showed that CEC can be modified by methotrexate (MTX) but not MTX + adalimumab (ADA) in RA patients [23]. A study reported in 2016 showed that the net cholesterol efflux did not change after RA therapy [25]. Since then, 11 population-based studies have been published, reporting inconsistent results.

Several potential mechanisms can thus be proposed to explain the changes in CEC, plasma HDL-C and CRP levels in RA patients. RA is an autoimmune disease associated with chronic inflammation, which might lead to multiple changes in the HDL structure and function [39]. First, some patients with RA may have elevated levels of certain proinflammatory cytokines, including CRP, interleukin-6 in their bloodstream. Many studies have indicated that HDL-C levels were dramatically reduced during inflammation, and one possible mechanism underlying these changes was associated with the liver phospholipid transfer protein (PLTP) expression. Of note, Audo et al. showed that PLTP was overexpressed in the joints of patients with RA, causing active inflammation [40]. Furthermore, Jiang et al. revealed that the plasma HDL-C levels was inversely associated with the plasma PLTP activity, and the surface components of triglyceride-rich lipoproteins play a positive role in keeping normal levels of HDL [41], which demonstrated that the inflammatory status and RA disease severity both were inversely related to the plasma HDL-C levels. However, the plasma HDL-C levels did not change significantly after RA treatment.

Secondly, substantial evidence has now accumulated suggesting a high inflammation status can limit the capacity for HDL to promote cholesterol efflux from macrophages [42]. McGillicuddy et al. conducted a study in mice found that RCT process was impaired by acute inflammation, decreasing HDL acceptor function, cholesterol efflux capacity, and cholesterol elimination [43]. Thus, the inflammatory status in patients with RA not only impair the rate of cholesterol efflux but also affect other processes in RCT. Prior studies have indicated that the circulating myeloperoxidase level is positively associated with the levels of oxidative stress and inflammation [44–46]. Furthermore, Vivekanandan-Giri reported that levels of myeloperoxidase-oxidized HDL were increased, and CEC was diminished in individuals with RA due to inflammation and oxidative stress [18]. Surprisingly, in the meta-analysis, CEC did not significantly differ between the RA and control subjects. It is possible that the conventional assays to measure CEC in RA patients may not be accurate due to fluctuations in the inflammatory status. Several studies used a mix of different approaches to measure CEC, which might partially result in underestimating the actual cholesterol efflux. However, a large quantity of population-based evidence has demonstrated that CEC is a sensitive predictor of the risk of CVD, regardless of the circulating HDL-C concentration [47, 48]. Besides, CEC significantly increases in patients with RA after medical care, followed by a reduction in the CRP level compared to the baseline, while the HDL-C level does not significantly change after anti-rheumatoid treatment. These findings indirectly indicate that CEC might be a more sensitive indicator than the HDL-C level in the prevention of CVDs in individuals with RA. Generally, effective treatment of inflammation through adequate RA disease control might help improve the function of HDL. Some possible mechanisms have been proposed to determine the specific role of elevating CEC in preventing CVDs in patients with RA. Therefore, additional larger scale population-based studies and experimental studies are needed to confirm these findings.

Heterogeneity poses a significant challenge to the conduction and interpretation of these meta-analysis results [49]. Subgroup analysis was conducted to evaluate the heterogeneity in the CEC and HDL-C levels, which showed that age was the source of statistical heterogeneity in HDL levels. 95% PI was also computed to assess the inter-study heterogeneity, which have found that the results of HDL-C and CRP included the null value leading to a decrease of the overall confidence in the evidence. Sensitivity analysis was conducted to enhance the robustness and reliability of the outcomes. There is no significant decrease in HDL-C levels in patients with RA when two studies were excluded [21, 22]. Meanwhile, the credibility ceilings test as a skeptical approach was applied to address the problem of spurious precision that accompanies the outcomes of observational studies, which could find a corrected summary effect estimate and probe the impact of added skepticism on conclusions. In this study, the results of CEC, CRP and ESR remained statistically significant, except for HDL-C levels with a 10% credibility ceiling. This was reflected also in the predictive interval calculated for HDL-C. However, all statistically significant findings did not survive at a 20% ceiling, which may indicate these results were uncertain. Therefore, these outcomes from observational studies should be viewed with some skepticism because of other hints of biases.

### Study strength and limitations

Several potential limitations should be taken into consideration. First, the quality of the meta-analyses depends largely on the quality of the original studies. Among the studies included, six observational studies were susceptible to selection and recall bias. Second, substantial heterogeneity was reported in this meta-analysis. Therefore, sensitivity analyses were conducted to confirm the stability of the results as well. Third, the small number of included studies might limit the ability to interpret the funnel plot. Fourth, subgroup analysis failed to perform by different treatment due to the small sample size, which might be the possible sources of heterogeneity. Finally, the most important limitation of this study is currently no established golden-standard assay for ex vivo evaluation of CEC. The choice of cholesterol acceptor can have a significant impact on the rate CEC, which might serve as the largest source of variation across studies. Although the standardized effect size was used to minimize the variation, it cannot eliminate methodological heterogeneity completely. Hence, these disadvantages possibly limit its widespread application.

## Conclusions

The current meta-analysis demonstrated that HDL-mediated CEC can be improved by the early control of inflammation and anti-rheumatic treatment in RA patients but does not affect the HDL-C levels. These findings and interpretations are limited by the quality and quantity of available evidence. Future randomized controlled trials with larger sample sizes and consensus-based methodologies are needed to determine the role of CEC in predicting CVD events in individuals with RA and whether anti-inflammatory therapeutic strategies to enhance CEC in individuals with RA have beneficial effects in preventing CVDs.

## Supplementary Information

**Additional file 1.** Forest plot of the plasma levels of LDL (a), TC (b) and TG (c) for patients with RA versus control group in observational study.

**Additional file 2.** Forest plot of the plasma levels of LDL-C (a), TC (b) and TG (c) for patients with RA and control group in before -after studies.

**Additional file 3.** Stratified analyses on the level of cholesterol efflux capacity in RA.

**Additional file 4.** Stratified analyses on the level of high-density lipoprotein in RA.

**Additional file 5.** Stratified analyses on the level of C-reactive protein in RA.

**Additional file 6.** Sensitivity analysis of the meta-analysis comparing the random and fixed models.

**Additional file 7.** Influence of each study on CEC, HDL-C, CRP and ESR in RA.

**Additional file 8.** Sensitivity analysis using credibility ceilings of 10% for the meta-analyses investigating the effect in RA.

**Additional file 9.** GRADE assessment of the systematic review and meta-analysis of observational studies assessing the effect of rheumatoid arthritis.

**Additional file 10.** GRADE assessment of the systematic review and meta-analysis of randomised trials assessing the effect of rheumatoid arthritis.

## Data Availability

Not applicable.

## Abbreviations

RA: Rheumatoid arthritis
CEC: Cholesterol efflux capacity
CVD: Cardiovascular diseases
OS: Oxidative stress
MPO: Myeloperoxidase
HDL-C: High-density lipoprotein cholesterol
TC: Total cholesterol
LDL-C: Low-density lipoprotein cholesterol
TG: Triglycerides
RCT: Reverse cholesterol transport
ESR: Erythrocyte sedimentation rate
MTX: Methotrexate
ADA: Adalimumab
PLTP: Phospholipid transfer protein
DAS28: Disease Activity Score for 28 joints
CRP: C-reactive protein
D&B: Downs and Black scale
NOS: Newcastle-Ottawa Scale
SMD: Standardized mean differences
MD: Mean differences
SD: Standard deviation
95%CIs: 95% Confidence intervals
95%PI: 95% Pretective intervals
