# Supplementary material for: A meta-analysis of HDL cholesterol efflux capacity and concentration in patients with rheumatoid arthritis
Source: Lipids Health Dis. 2021 Feb 21;20:18. doi: 10.1186/s12944-021-01444-6 (PMC7897392; doi:10.1186/s12944-021-01444-6)
Supplement: Supplementary file 1 — Additional file 1. Forest plot of the plasma levels of LDL (a), TC (b) and TG (c) for patients with RA versus control group in observational study. [file 12944_2021_1444_MOESM1_ESM.docx]

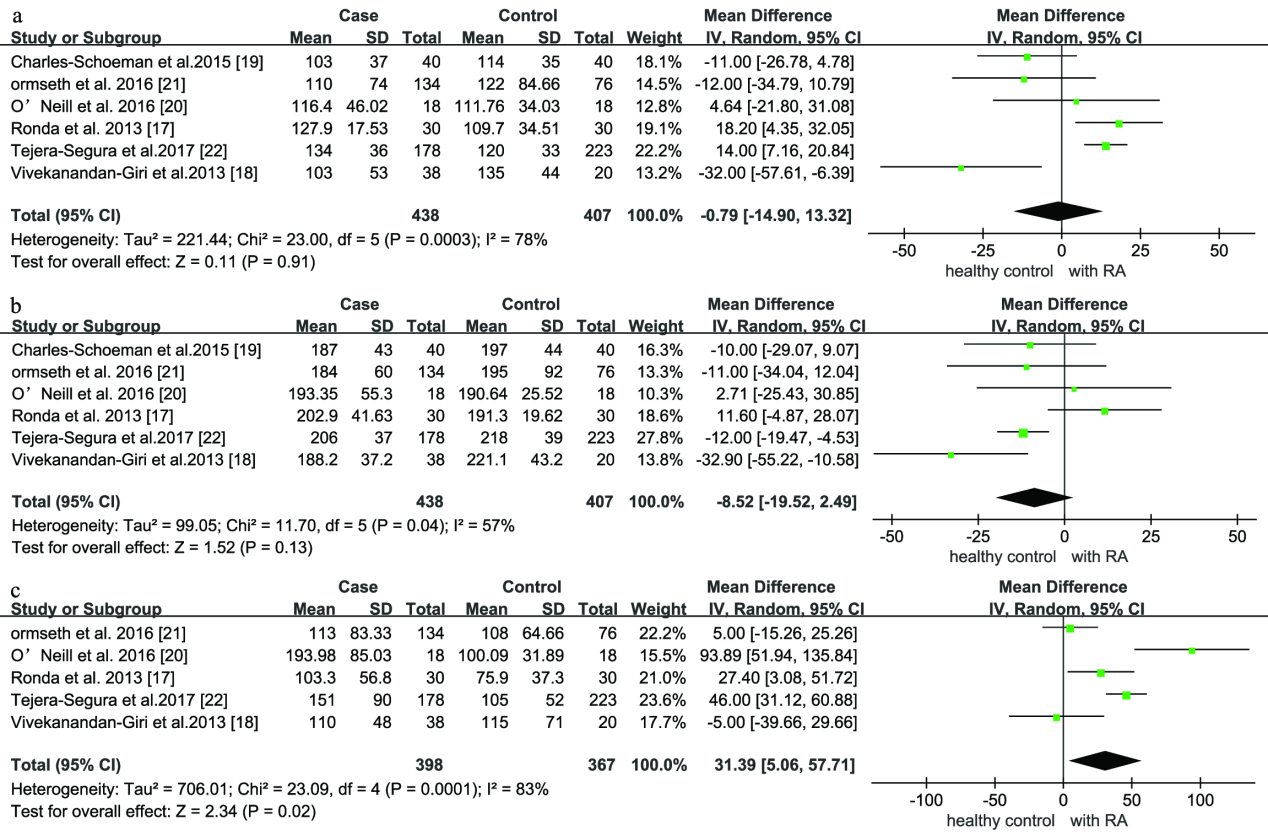
**Additional file 1.** Forest plot of the plasma levels of LDL (a), TC (b) and TG (c) for patients with RA versus control group in observational study.
