# Supplementary material for: A meta-analysis of HDL cholesterol efflux capacity and concentration in patients with rheumatoid arthritis
Source: Lipids Health Dis. 2021 Feb 21;20:18. doi: 10.1186/s12944-021-01444-6 (PMC7897392; doi:10.1186/s12944-021-01444-6)
Supplement: Supplementary file 3 — Additional file 3. Stratified analyses on the level of cholesterol efflux capacity in RA. [file 12944_2021_1444_MOESM3_ESM.docx]

| **Subgroups** | **No. of patients** | **No. of studies** | **SMD (95% CI)^a^** | ***P*^b^** | **Heterogeneity** | |
| --- | --- | --- | --- | --- | --- | --- |
|  |  |  |  |  | ***I*^2^ (%)** | ***P* ^c^** |
| DAS28^d^ |  |  |  |  |  |  |
| < 2.6 | 58 | 1 | -1.49 (-2.10, -0.89) | < 0.001 | - | - |
| ≥ 2.6 | 751 | 4 | -0.10 (-0.47, 0.27) | 0.600 | 81.0 | 0.001 |
| Age |  |  |  |  |  |  |
| < 55 | 350 | 3 | -0.24 (-0.58, 0.08) | 0.150 | 52.5 | 0.122 |
| ≥ 55 | 459 | 2 | -0.34 (-2.29, 1.05) | 0.468 | 96.3 | < 0.001 |
| Study design |  |  |  |  |  |  |
| Case-control | 198 | 3 | -0.54 (-1.41, 0.34) | 0.229 | 88.4 | 0.525 |
| Cross-sectional | 620 | 2 | -0.13 (-0.79, 0.54) | 0.715 | 93.3 | 0.217 |

**Additional file 3.** Stratified analyses on the level of cholesterol efflux capacity in RA

**The results of stratified analyses were generated from the analyses comparing highest vs. lowest group.**

**^a^ SMD and 95%CIs; ^b^ *P*-value of Z-test for the significance of the SMD and 95%CIs; ^c^ *P*-value for heterogeneity; ^d^ Activity Score for 28 joints**
