# Supplementary material for: A meta-analysis of HDL cholesterol efflux capacity and concentration in patients with rheumatoid arthritis
Source: Lipids Health Dis. 2021 Feb 21;20:18. doi: 10.1186/s12944-021-01444-6 (PMC7897392; doi:10.1186/s12944-021-01444-6)
Supplement: Supplementary file 4 — Additional file 4. Stratified analyses on the level of high-density lipoprotein in RA. [file 12944_2021_1444_MOESM4_ESM.docx]

**Additional file 4.** Stratified analyses on the level of high-density lipoprotein in RA

**The results of stratified analyses were generated from the analyses comparing highest vs. lowest group.**

**^a^ WMD and 95%CIs; ^b^ *P*-value of Z-test for the significance of the WMD and 95%CIs; ^c^ *P*-value for heterogeneity; ^d^ Activity Score for 28 joints.**

| **Subgroups** | **No. of patients** | **No. of studies** | **WMD (95% CI)^a^** | ***P^b^*** | **Heterogeneity** | |
| --- | --- | --- | --- | --- | --- | --- |
|  |  |  |  |  | ***I*^2^ (%)** | ***P***^c^ |
| DAS28^d^ |  |  |  |  |  |  |
| < 2.6 | 58 | 1 | -3.00 (-11.91, 5.91) | 0.509 | - | - |
| ≥ 2.6 | 787 | 5 | -3.98 (-7.62, -0.35) | 0.121 | 70.8 | 0.008 |
| Age |  |  |  |  |  |  |
| < 55 | 350 | 3 | -1.42 (-5.06, 2.21) | 0.442 | 35.0 | 0.215 |
| ≥ 55 | 495 | 3 | -7.34 (-11.14, -3.54) | < 0.001 | 19.6 | 0.288 |
| Study design |  |  |  |  |  |  |
| Case-control | 234 | 4 | -2.81 (-9.80, 4.16) | 0.430 | 62.7 | 0.525 |
| Cross-sectional | 620 | 2 | -3.91 (-7.15, -0.68) | 0.018 | 82.0 | 0.217 |
