# Supplementary material for: A meta-analysis of HDL cholesterol efflux capacity and concentration in patients with rheumatoid arthritis
Source: Lipids Health Dis. 2021 Feb 21;20:18. doi: 10.1186/s12944-021-01444-6 (PMC7897392; doi:10.1186/s12944-021-01444-6)
Supplement: Supplementary file 5 — Additional file 5. Stratified analyses on the level of C-reactive protein in RA. [file 12944_2021_1444_MOESM5_ESM.docx]

| **Subgroups** | **No. of patients** | **No. of studies** | **SMD (95% CI)^a^** | ***P*^b^** | **Heterogeneity** | |
| --- | --- | --- | --- | --- | --- | --- |
|  |  |  |  |  | ***I^2^* (%)** | ***P*^c^** |
| Age |  |  |  |  |  |  |
| < 55 | 350 | 3 | 1.38 (-0.17, 2.93) | 0.080 | 97.2 | < 0.001 |
| ≥ 55 | 446 | 2 | 4.96 (3.04, 6.88) | < 0.001 | 82.4 | 0.017 |
| Study design |  |  |  |  |  |  |
| Case-control | 176 | 3 | 2.19 (0.41, 3.97) | 0.016 | 95.4 | < 0.001 |
| Cross-sectional | 620 | 2 | 3.50 (2.26, 4.74) | < 0.001 | 95.5 | < 0.001 |

**Additional file 5.** Stratified analyses on the level of C-reactive protein in RA

**The results of stratified analyses were generated from the analyses comparing highest vs. lowest group.**

**^a^ SMD and 95%CIs; ^b^ *P*-value of Z-test for the significance of the SMD and 95%CIs. ^c^ *P*-value for heterogeneity.**
