# Supplementary material for: A meta-analysis of HDL cholesterol efflux capacity and concentration in patients with rheumatoid arthritis
Source: Lipids Health Dis. 2021 Feb 21;20:18. doi: 10.1186/s12944-021-01444-6 (PMC7897392; doi:10.1186/s12944-021-01444-6)
Supplement: Supplementary file 6 — Additional file 6. Sensitivity analysis of the meta-analysis comparing the random and fixed models. [file 12944_2021_1444_MOESM6_ESM.docx]

**Additional file 6.** Sensitivity analysis of the meta-analysis comparing the random and fixed models

| **Comparison** | **Random effects model** | | |  | **Fixed effects model** | | |
| --- | --- | --- | --- | --- | --- | --- | --- |
|  | **Effect size** | **95% CI^e^** | ***P*-value** |  | **Effect size** | **95% CI** | ***P*-value** |
| CEC^a^ | -0.34* | -0.86, 0.14 | 0.170 |  | -0.10* | -0.24, 0.04 | 0.160 |
| HDL^b^ | -3.91** | -7.15, -0.68 | 0.020 |  | -5.21** | -7.69, -2.72 | < 0.001 |
| CRP^c^ | 2.74* | 1.13, 4.36 | < 0.001 |  | 2.53* | 2.33, 2.74 | < 0.001 |
| ESR^d^ | 1.22 | 0.66, 1.78 | < 0.001 |  | 1.39* | 1.20,1.58 | < 0.001 |

***: Standard mean difference; **: mean difference; ^a^cholesterol efflux capacity.**

**^b^ high-density lipoprotein; ^c^C-reactive protein; ^d^erythrocyte sedimentation rate; ^e^CI: Confidence interval.**
