# Supplementary material for: A meta-analysis of HDL cholesterol efflux capacity and concentration in patients with rheumatoid arthritis
Source: Lipids Health Dis. 2021 Feb 21;20:18. doi: 10.1186/s12944-021-01444-6 (PMC7897392; doi:10.1186/s12944-021-01444-6)
Supplement: Supplementary file 7 — Additional file 7. Influence of each study on CEC, HDL-C, CRP and ESR in RA. [file 12944_2021_1444_MOESM7_ESM.docx]

**Additional file 7.** Influence of each study on CEC, HDL-C, CRP and ESR in RA

| **Study omitted** | **SWD^a^/MD^b^** | **Lower limit of 95% CI^c^** | **Upper limit of 95% CI** |  |
| --- | --- | --- | --- | --- |
| **CEC^d^** |  |  |  | |
| Charles-Schoeman et al, 2015 [19] | -0.45 | -1.05 | 0.15 | |
| Ormseth et al, 2016 [21] | -0.32 | -0.94 | 0.31 | |
| Ronda et al, 2013[17] | -0.37 | -0.96 | 0.21 | |
| Tejera-Segura et al, 2017 [22] | -0.50 | -1.02 | 0.03 | |
| Vivekanandan-Giri et al, 2013 [18] | -0.10 | -0.48 | 0.28 | |
| **HDL-C^e^** |  |  |  | |
| Charles-Schoeman et al, 2015 [19] | -4.50 | -8.03 | -0.96 | |
| Ormseth et al, 2016 [21] | -4.04 | -9.16 | 1.08 | |
| O’Neill et al, 2016 [20] | -3.15 | -6.05 | -0.26 | |
| Ronda et al, 2013 [17] | -4.76 | -8.11 | -1.42 | |
| Tejera-Segura et al, 2017 [22] | -2.85 | -6.85 | 1.14 | |
| Vivekanandan-Giri et al, 2013 [18] | -3.99 | -7.62 | -0.35 | |
| **CRP^f^** |  |  |  | |
| Charles-Schoeman et al, 2015 [19] | 3.25 | 1.61 | 4.96 | |
| Ormseth et al, 2016 [21] | 2.76 | 0.52 | 4.99 | |
| O’Neill et al, 2016 [20] | 2.06 | 0.33 | 3.79 | |
| Ronda et al, 2013 [17] | 3.28 | 1.50 | 5.05 | |
| Tejera-Segura et al, 2017 [22] | 2.34 | 0.76 | 3.92 | |
| **ESR^g^** |  |  |  | |
| Charles-Schoeman et al, 2015 [19] | 1.54 | 1.33 | 1.75 | |
| Ronda et al, 2013[17] | 1.16 | 0.31 | 2.02 | |
| Tejera-Segura et al, 2017 [22] | 0.98 | 0.39 | 1.57 | |

**^a^ standardized mean differences; ^b^ weighted mean differences; ^c^ confidence interval; ^d^ cholesterol efflux capacity; ^e^ high-density lipoprotein cholesterol; ^f^ C-reactive protein levels; ^g^ erythrocyte sedimentation rate.**
