# Supplementary material for: A meta-analysis of HDL cholesterol efflux capacity and concentration in patients with rheumatoid arthritis
Source: Lipids Health Dis. 2021 Feb 21;20:18. doi: 10.1186/s12944-021-01444-6 (PMC7897392; doi:10.1186/s12944-021-01444-6)
Supplement: Supplementary file 8 — Additional file 8. Sensitivity analysis using credibility ceilings of 10% for the meta-analyses investigating the effect in RA. [file 12944_2021_1444_MOESM8_ESM.docx]

**Additional file 8.** Sensitivity analysis using credibility ceilings of 10% for the meta-analyses investigating the effect in RA

| **Study ID** | **outcome** | **10% Credibility ceiling** | | | **20% Credibility ceiling** | | |
| --- | --- | --- | --- | --- | --- | --- | --- |
|  |  | **ES^a^ (95% CI^b^)** | ***I^2^* (%)** | **LR^c^** | **ES (95% CI)** | ***I^2^* (%)** | **LR** |
| Charles-Schoeman et al, 2015 [19]  Ormseth et al, 2016 [21]  Ronda et al, 2013 [17]  Tejera-Segura et al, 2017 [22]  Vivekanandan-Giri et al, 2013 [18] | CEC^d^ | -0.04 (-0.33, 0.25) | 31.8 | 0.29 | -0.01(-0.27, 0.26) | 0 | 0.01 |
| Charles-Schoeman et al, 2015 [19]  Ormseth et al, 2016 [21]  O’Neill et al, 2016 [20]  Ronda et al, 2013 [17]  Tejera-Segura et al, 2017[22]  Vivekanandan-Giri et al, 2013 [18] | HDL-C^e^ | -2.90 (-5.24, 1.05) | 0 | 4.22 | -1.13 (-4.99, 2.71) | 0 | 0.78 |
| Charles-Schoeman et al, 2015 [19]  Ormseth et al, 2016 [21]  O’Neill et al, 2016 [20]  Ronda et al, 2013 [17]  Tejera-Segura et al, 2017 [22] | CRP^f^ | 0.74 (0.08, 1.40) | 0 | 34.66 | 0.74 (-0.27, 1.75) | 0 | 5.71 |
| Charles-Schoeman et al, 2015 [19]  Ronda et al, 2013 [17]  Tejera-Segura et al, 2017 [22] | ESR^g^ | 0.93 (0.05, 1.81) | 0 | 25.76 | 0.93 (-0.41, 2.27) | 0 | 4..83 |

**^a^effect size; ^b^confidence interval; ^c^Likelihood Ratio for the corresponding credibility ceiling;**

**^d^cholesterol efflux capacity; ^e^high-density lipoprotein cholesterol; ^f^c-reactive protein; ^g^erythrocyte sedimentation rate.**
