# Supplementary material for: A meta-analysis of HDL cholesterol efflux capacity and concentration in patients with rheumatoid arthritis
Source: Lipids Health Dis. 2021 Feb 21;20:18. doi: 10.1186/s12944-021-01444-6 (PMC7897392; doi:10.1186/s12944-021-01444-6)
Supplement: Supplementary file 9 — Additional file 9. GRADE assessment of the systematic review and meta-analysis of observational studies assessing the effect of rheumatoid arthritis. [file 12944_2021_1444_MOESM9_ESM.docx]

**Additional file 9.** GRADE assessment of the systematic review and meta-analysis of observational studies assessing the effect of rheumatoid arthritis

| The effect of rheumatoid arthritis | No. of  studies | Design | Limitations | Inconsistency | Indirectness | Imprecision | Other considerations | Quality |
| --- | --- | --- | --- | --- | --- | --- | --- | --- |
| CEC | 5 | observational studies | no serious limitations | serious^1^ | no serious indirectness | no serious imprecision | None^5^ | ⊕OOO VERY LOW |
| HDL-C | 6 | observational studies | no serious limitations | no serious^2^ inconsistency | no serious indirectness | no serious imprecision | None^5^ | ⊕⊕OO LOW |
| CRP | 5 | observational studies | no serious limitations | serious^3^ | no serious indirectness | no serious imprecision | None^5^ | ⊕OOO VERY LOW |
| ESR | 3 | observational studies | no serious limitations | serious^4^ | no serious indirectness | no serious imprecision | None^5^ | ⊕OOO VERY LOW |

**^1^Serious inconsistency due to high heterogeneity (*I²* = 92%; *P* < 0.001).**

**^2^ Part of the heterogeneity might be explained by age and study design.
^3^Serious inconsistency due to high heterogeneity (*I²* = 98%; *P* < 0.001).**

**^4^Serious inconsistency due to high heterogeneity (*I²* = 83%; *P* < 0.001).**

**^5^Slight asymmetry was detected in the funnel plot, the results from the Egger’s and Begg’s tests did not show evidence of publication bias, however, there were <10 studies which may have not been enough power to distinguish chance from real funnel plot.**
