# Supplementary material for: A meta-analysis of HDL cholesterol efflux capacity and concentration in patients with rheumatoid arthritis
Source: Lipids Health Dis. 2021 Feb 21;20:18. doi: 10.1186/s12944-021-01444-6 (PMC7897392; doi:10.1186/s12944-021-01444-6)
Supplement: Supplementary file 10 — Additional file 10. GRADE assessment of the systematic review and meta-analysis of randomised trials assessing the effect of rheumatoid arthritis. [file 12944_2021_1444_MOESM10_ESM.docx]

**Additional file 10.** GRADE assessment of the systematic review and meta-analysis of randomised trials assessing the effect of rheumatoid arthritis

| The effect of rheumatoid arthritis | No. of  trails | Design | Limitations | Inconsistency | Indirectness | Imprecision | Other considerations | Quality |
| --- | --- | --- | --- | --- | --- | --- | --- | --- |
| CEC | 8^1^ | randomised trials | serious^2^ | no serious inconsistency | no serious indirectness | serious^3^ | none | ⊕⊕OO LOW |
| HDL-C | 5^4^ | randomised trials | serious^2^ | no serious inconsistency | no serious indirectness | serious^5^ | none | ⊕⊕OO LOW |
| CRP | 3 | randomised trials | serious^6^ | no serious inconsistency | no serious indirectness | no serious imprecision | none | ⊕⊕⊕O MODERATE |
| ESR | 3^7^ | randomised trials | serious^6^ | serious^8^ | no serious indirectness | serious^9^ | none | ⊕OOO  VERY LOW |

**^1^ There are 5 studies with 8 trails.
^2^ There are one randomized Controlled Trials and four semi-randomized.
^3^ The 95% CI (0.20, 0.37%) overlaps with the minimally important difference of 0.2%.
^4^ There are 4 studies with 5 trails.
^5^ The 95% CI (-0.13, 7.39mg/dL) overlaps with the minimally important difference of 3.87mg/dL.
^6^ There are one randomized Controlled Trials and two semi-randomized.
^7^ There are 3 studies with 5 trails.
^8^ Serious inconsistency due to high heterogeneity (*I²* = 92%; *P* < 0.001).
^9^ Although the 95% CI (--3.23, -0.74mm/h) does not include the minimally important difference of 3.87mm/h, neither the upper or lower bound of the 95% CI are lower or higher than 3.87mm/h, respectively.**
